# Vibration Monitoring Using Fiber Optic Sensors in a Lead-Bismuth Eutectic Cooled Nuclear Fuel Assembly †

**DOI:** 10.3390/s16040571

**Published:** 2016-04-21

**Authors:** Ben De Pauw, Alfredo Lamberti, Julien Ertveldt, Ali Rezayat, Katrien van Tichelen, Steve Vanlanduit, Francis Berghmans

**Affiliations:** 1 Brussels Photonics Team (B-Phot), Vrije Universiteit Brussel, Pleinlaan 2, 1050 Brussels, Belgium; fberghma@vub.ac.be; 2 Acoustics and Vibration Research Group, Vrije Universiteit Brussel, Pleinlaan 2, 1050 Brussels, Belgium; allamber@vub.ac.be (A.L.); julien.ertveldt@vub.ac.be (J.E.); arezayat@vub.ac.be (A.R.); Steve.Vanlanduit@uantwerpen.be (S.V.); 3 Belgian Nuclear Research Centre (SCK·CEN), Boeretang 200, Mol, Belgium; katrien.van.tichelen@sckcen.be

**Keywords:** liquid metal flows, FBG, vibration, operational modal analysis, experiments, fluid-structure interaction, high temperature

## Abstract

Excessive fuel assembly vibrations in nuclear reactor cores should be avoided in order not to compromise the lifetime of the assembly and in order to prevent the occurrence of safety hazards. This issue is particularly relevant to new reactor designs that use liquid metal coolants, such as, for example, a molten lead-bismuth eutectic. The flow of molten heavy metal around and through the fuel assembly may cause the latter to vibrate and hence suffer degradation as a result of, for example, fretting wear or mechanical fatigue. In this paper, we demonstrate the use of optical fiber sensors to measure the fuel assembly vibration in a lead-bismuth eutectic cooled installation which can be used as input to assess vibration-related safety hazards. We show that the vibration characteristics of the fuel pins in the fuel assembly can be experimentally determined with minimal intrusiveness and with high precision owing to the small dimensions and properties of the sensors. In particular, we were able to record local strain level differences of about 0.2 μϵ allowing us to reliably estimate the vibration amplitudes and modal parameters of the fuel assembly based on optical fiber sensor readings during different stages of the operation of the facility, including the onset of the coolant circulation and steady-state operation.

## 1. Introduction

**Figure 1:**
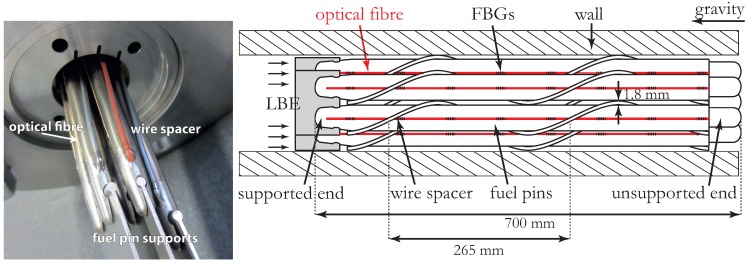
Concept drawing and photograph of the seven-pin fuel assembly.

This paper pertains to research activities conducted with the aim to develop and to commission the Multi-Purpose Hybrid Research Reactor for High-tech Applications (MYRRHA), which is a prototype nuclear reactor belonging to the so-called fourth generation [1]. MYRRHA will be an accelerator driven and spallation source based reactor cooled with molten lead-bismuth eutectic (LBE). As in any nuclear reactor, the vibration levels of reactor internals have to be kept within certain limits. Excessive vibrations of reactor components have caused several failures in the past, as discussed in [2]. In order to determine the damage mechanisms related to vibrations and to understand how these may reduce the lifetime of the reactor components, one requires accurate measurements of the vibration amplitudes and modal parameter [3,4]. One particularly important reactor component is the fuel assembly. In MYRRHA, one fuel assembly consists of a total of 127 wire spaced parallel fuel pins. Figure 1 shows a sub-assembly consisting of seven fuel pins. These fuel pins are placed vertically in the reactor and are mechanically supported only at the bottom. The top remains essentially free to move. A wire spacer is wrapped around every fuel pin to ensure that they remain properly spaced during operation. The LBE coolant is flowing upwards with flow speed around 2 m/s and interacts with the fuel assembly. This fluid-structure interaction causes the fuel assembly to vibrate.

Different optical and electro-mechanical vibration sensors or techniques have already been successfully applied to measure coolant-flow induced vibration in a nuclear fuel assembly [5,6,7]. Heavy liquid metal coolants have a relatively high density flow at elevated temperature (300 ${}^{{}^{\circ}}$C in our case) and are corrosive and opaque. This shortens the list of potentially adequate sensors or techniques as, for example, piezoelectric sensors would suffer from electrode corrosion or require bulky packaging to protect the sensors. In our application, the spacing between the individual fuel pins is limited to a couple of millimeters, which leaves little room to install vibration sensors. Furthermore, and in order to obtain reliable vibration readings, the sensors should not affect the flow of LBE. Therefore, the selected sensors should have very small dimensions. This led us to select optical fiber Bragg grating (FBG) based sensors which we have validated for this specific environment in [8,9]. Other optical fiber based sensors such as distributed sensing are not considered in this application because these technologies do not meet the acquisition speed and precision required for the vibration measurement (as also explained later).

In this paper, we demonstrate actual vibration measurements of an LBE cooled fuel assembly. To do so, we instrumented a seven-pin scaled mock-up of the MYRRHA fuel assembly (Figure 1) and subjected it to a flow of LBE. We evaluated the vibration amplitudes and we identified the modal parameters for several LBE flow conditions. To the best of our knowledge, FBG sensors have never been applied for this purpose. Note that the objective is to determine the vibrations in a mock-up situation, with the eventual intention to support the optimization of the design of the fuel assembly. Consequently, the effects of nuclear radiation on the FBG sensors do not need to be taken into account in this study. Note also that the influence of highly energetic radiation on the flow-induced vibrations themselves can be considered to be a secondary effect.

## 2. Experimental Set-Up

### 2.1. Instrumentation of the Fuel Assembly

We first fabricated a mock-up fuel assembly consisting of seven fuel pins. These fuel pins are made from stainless steel (316 L) cylindrical tubes with a diameter of 6.55 mm and a length of 700 mm (Figure 1). The diameter corresponds to that of the actual pins that will be used in MYRRHA, whilst the length is only half of the actual value. The fuel pins are wrapped with a wire spacer (diameter 1.8 mm) in a helical fashion with a pitch of 265 mm. In MYRRHA, the fuel pins will be arranged in the fuel assembly in a hexagonal lattice and the vessel wall will tightly enclose this assembly. To mimic the situation anticipated in MYRRHA, a hexagon-shaped shell was placed around the fuel bundle.

Due to the limited space of 1.8 mm between the individual fuel pins and between the fuel pins and the vessel wall, optical fiber sensors are one of the very few options to measure vibration in the fuel assembly. As mentioned above, we have already shown that, first, FBG based sensors are sufficiently sensitive to pick up the fuel pin vibration [10,11], and, second, their useful time in an LBE environment is sufficient to carry out meaningful measurements [8,9]. More specifically, we demonstrated that FBGs pick up fuel pin vibrations with displacements as small as 10 to 60 μm which are representative for fuel pin bending modes and compared the results with conventional electro-mechanical and optical sensors or techniques. This benchmark evidenced that FBGs are the most adequate sensors to pick up fuel pin vibration given the experimental conditions. We also assessed the useful time of FBGs in an LBE environment by exposing instrumented fuel pins to molten lead-bismuth and by continuously monitoring the spectral response. This indicated that vibration measurements yielding wavelength shifts on the order of 1 pm can be carried out reliably during at least 700 h in LBE.

**Figure 2:**
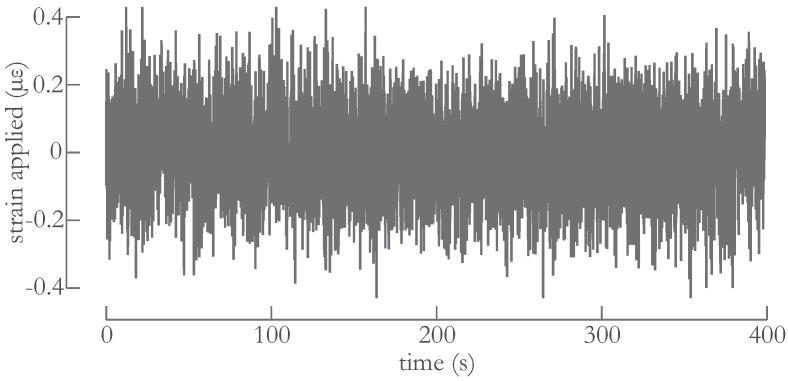
Owing to the phase-correlation approach to demodulation, the reflected spectral information of the FBGs and the (temperature) controlled environment, the strain resolution (determined as the standard deviation of the noise) was approximately 0.2 μϵ.

For sake of completeness, we briefly recall the principle of operation of an FBG based sensor. An FBG consists of a periodic variation of the refractive index of the core of an optical fiber and is fabricated using dedicated laser-based techniques (see [12,13] for more details). The reflection spectrum of an FBG is characterized by a resonance center wavelength at which it reflects the largest optical power. This wavelength is referred to as the Bragg wavelength $\lambda_{B}$. This $\lambda_{B}$ depends on the strain or temperature applied to the FBG. Tracking the value of $\lambda_{B}$ as a function of time therefore allows using the FBG as a sensor. We acquired the spectra of the FBGs using a commercially FBG-Scan 704D (FBGS Int. Geel, Belgium) [14], which is a high-speed FBG sensor interrogation unit. Since the expected wavelength fluctuation is small (as illustrated in [15]) and signal degradation due to the environment is expected to occur (as explained in more detail in [8,9]), we opted for a phase-correlation based approach to analyse the acquired FBG spectra and to calculate $\lambda_{B}$ [16]. This approach allowed achieving the best trade-off between signal-to-noise ratio and calculation speed even if the FBG spectra become distorted. Owing to the use of the phase-correlation based approach and to the temperature controlled environment (explained in detail in the next section), we were able to record Bragg wavelength shifts as small as 0.25 pm corresponding to a local strain level difference of about 0.2 μϵ. This resolution is demonstrated in Figure 2, which shows the measured applied strain for a single FBG when all air is removed from the set-up, as explained later. The FBG spectra were recorded and analyzed in real-time with an acquisition rate of almost 5000 measurements per second.

**Figure 3:**
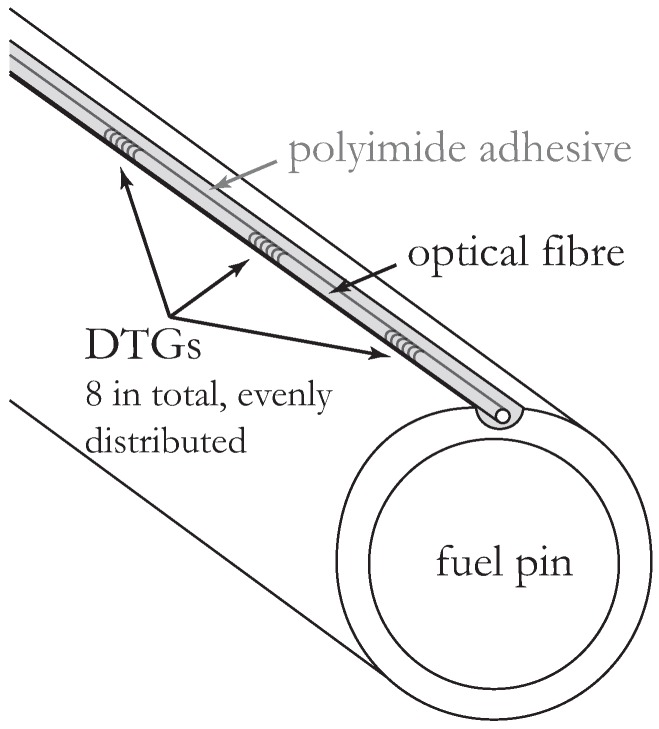
Scheme of an optical fiber with draw tower gratings (DTGs) embedded in a small groove near the surface of the pin.

In order to perform reliable vibration measurements on the surface of the individual fuel pins within the fuel assembly, the FBGs need to be adequately mounted. To do so, we followed the procedure explained in [8,9] and illustrated in Figure 3, in which we show a fuel pin equipped with an FBG. The optical fiber, which is only 200 μm in diameter, is inserted within a small groove that has been engraved (using electrical discharge machining (EDM)) at the fuel pin surface. Using this approach, the gap between the individual fuel pins is kept clear. The fiber is then fixed in the groove with a high temperature (>300 ${}^{{}^{\circ}}$C) polyimide adhesive (Polytec TC-P490) (Polytec PT, Waldbronn, Germany) which is rated for continuous operation up to 400 ${}^{{}^{\circ}}$C. Upon specific temperature curing, it sets to provide stiff adhesion (shore hardness of D75 and Young’s modulus > 5 GPa) between the optical fiber and the fuel pin structure. The substance has adequate out-gassing properties and can serve as a non-abrasive filler while retaining excellent chemical and moisture resistance. The adherence of this adhesive to both glass and stainless steel is excellent. Note that, after curing of the adhesive, we kept the instrumented fuel pin at 275 ${}^{{}^{\circ}}$C for several hours in order to (further) anneal the gratings. As explained in more detail in [8], this integration method allowed us to obtain a strain transfer from the fuel pin to the optical fiber exceeding 85% without any significant distortion in the reflection spectrum of the FBGs. The FBGs are commercially available draw tower gratings (DTGs) [14,17,18], which are known to feature excellent strength and fatigue characteristics. The former property is especially important during thermal cycles (and shocks), where we encountered corresponding strains as high as 1% due to thermal expansion. The higher yield strength of DTGs is also particularly important during installation of the fuel pins in the complex experiments; as they feature a higher strength than conventional re-coated FBGs, they are less likely to fail during installation and use.

Every fuel pin of the seven-pin assembly was instrumented with an optical fiber. We guided each fiber outside the loop through a pair of stainless steel tubes which were loosely inserted in one another (not shown in Figure 1 for sake of clarity), so as not to affect the movement of the fuel pins because of instrumentation. Each fiber contained eight FBGs uniformly distributed over the fuel pin length as illustrated in Figure 1, Figure 2 and Figure 3. Some of the fibers had one extra FBG that was not mounted on the fuel pin but was located in the stainless steel tubes close to the fuel pin extremity. The function of this extran FBG is explained later in the modal analysis. We determined that eight FBGs suffice to conduct the modal analysis. In addition, the uniform distribution of these sensors facilitates a more straightforward modal analysis. This fuel assembly was then inserted in a set-up that could circulate LBE whilst keeping the LBE temperature constant and uniform. This, in combination with high-pass filtering explained later, allowed removing the effects of temperature variations on the FBG readings.

### 2.2. Experimental Test Loop

**Figure 4:**
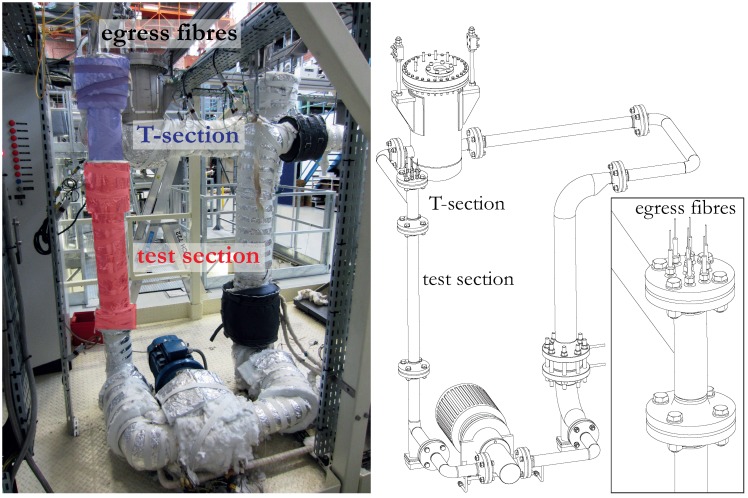
Photograph and scheme of the test facility containing the fuel assembly at SCK·CEN. The inset shows an enlarged image of the T-section holding the egress locations of the optical fibers.

We have constructed a loop to circulate LBE in order to expose the instrumented fuel assembly to a flow of LBE in conditions that mimic those anticipated in the MYRRHA reactor. Figure 4 shows the full set-up, which measures 3.5 m by 2.5 m and which consists of an LBE reservoir, the actual test loop and the control systems. The loop can hold around 350 kg of LBE at temperatures up to 250 ${}^{{}^{\circ}}$C. A 5.5 kW motor (type FCA 132SA-2) (AC-Motoren, Eppertshausen, Germany) powers the magnetically coupled three screw pump (type Kral K 55-118) (Kral, Lustenau, Österreich) to achieve mass flow rates up to 20.5 kg/s. The heating and control system relies on a network of pressure sensors, tachometers and 35 independent heating circuits each having several heat tracing lines combined with k-type thermocouples mounted on the outer shell of the loop. Prior to the start of the pump, the loop is heated above the melting temperature of LBE (123 ${}^{{}^{\circ}}$C) and air is eliminated to prevent oxidation of the LBE. The loop is then filled with LBE and kept under argon flow at a slight overpressure (±1 bar extra) so as to avoid infiltration of oxygen. The vertical test section, located shortly after the pump, consists of a 0.767 m long steel cylinder with a 51.2 mm inner diameter and equipped with a 0.450 m T-section on top. The T-section diverts the LBE flow and also holds the egress locations of the optical fibers.

## 3. Results and Discussion

### 3.1. Vibration Amplitude Evaluation

**Figure 5:**
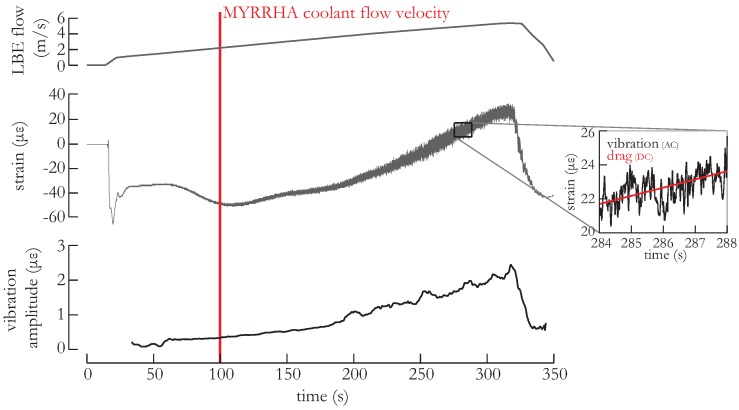
(**Top**): The LBE flow velocity during the course of the test procedure; middle: Corresponding average strain values as measured with *all* the FBGs on *all* fuel pins; (**Bottom**): rms value of the strain AC component of the middle figure; inset: Illustration of the high-pass filtering applied to the strain measurements to eliminate the drag (*i.e.*, the DC component (**red**)) and obtain the vibration (AC component).

The foremost important quantity required to assess potential safety hazards related to reactor component vibration is the vibration amplitude [3,4]. During exploitation of a nuclear reactor, many different operational conditions can occur in the coolant flow of a fuel assembly including different coolant flow velocities and transients during start-up. All these operational conditions should be investigated when studying the flow-induced vibration as the vibration amplitude in every conditions can differ significantly. We measured the dynamic strain using the FBGs on the surface of the fuel pins in steady flow and varying flow conditions as well as during transients e.g., when the pump is switched on. The procedure was as follows. We started from stagnant LBE, and we gradually increased the flow velocity up to nearly 6 m/s. Once the maximum flow velocity was reached, we stopped the pump. We illustrate the course of the procedure in Figure 5 with, from top to bottom, the LBE flow velocity, the average strain values as measured with all the FBGs on all fuel pins and these measurements processed with a high-pass filter so to obtain the rms value of the AC strain component. This high-pass filtering was essentially done to detrend the data and consisted of subtracting Savitzky–Golay filtered [19] data with a span of 20,000 samples *i.e.*, four seconds of measurement time from the unfiltered data. We then obtained the vibration amplitude for the filtered data by determining the rms values in a moving window with a width of one second (or 5000 samples). The largest vibration amplitude clearly occurred during the start-up of the pump. Note that the operation of the pump starts at approximately 0.6 m/s, generating a transient in the flow velocity. In this case, the vibration amplitude exceeded 70 μϵ. However, this transient disappears after a few seconds. We believe that the transient during the start-up of the pump is caused by the initial push of the flow on the fuel assembly resulting in a compressive force (see Figure 5 (middle)). When increasing the flow velocity we observed a corresponding increase in the vibration amplitude but also in the amount of drag on the fuel pins. In the inset of Figure 5, we illustrate that the amount of drag corresponds to the DC value (*i.e.*, the part that was subtracted with the high-pass filter) whilst the vibration amplitude corresponds to the AC value (*i.e.*, with the high-pass filter applied) of the unfiltered strain data. Recall that since the temperature of the LBE coolant is maintained with the temperature control system, the temperature fluctuations were minimal: temperature differences determined with the temperature control system were smaller than 0.1 ${}^{{}^{\circ}}$C across the test section. For LBE coolant flow velocities as anticipated in MYRRHA (indicated with the red line), we observed an average vibration amplitude close to 0.5 μϵ. At the highest tested flow velocity, we measure an vibration amplitude of nearly three μϵ. When the highest flow speed was achieved, we stopped the pump. A control loop managing the pump prevents the occurrence of excessively fast transients in the pump rotation and hence the shut-down was not instantaneous.

**Figure 6:**
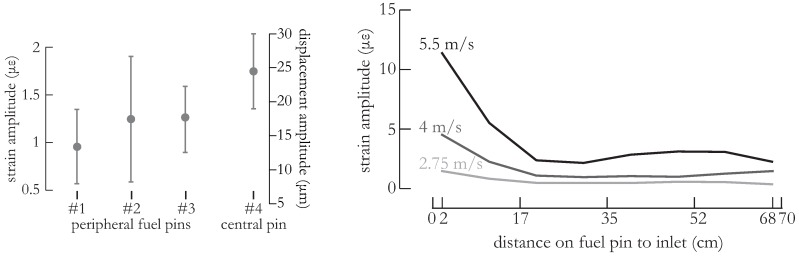
(**Left**): rms vibration amplitude and corresponding standard deviation averaged over all FBGs per fuel pin at an LBE flow of 4 m/s. The central fuel pin exhibits a slightly higher vibration amplitude compared to the peripheral fuel pins; (**Right**): The rms vibration amplitude measured with the different FBGs on the central fuel pin.

Next, we assessed the pin-to-pin vibration amplitude during normal flow conditions. In Figure 6 (left), we have determined the rms vibration amplitude in terms of strain averaged over all FBGs per fuel pin. Due to damaged fibers and to the limited amount of channels available on the FBG interrogation device, we limited the assessment to four fuel pins. In this figure, we have subjected the fuel assembly to an LBE flow of nearly 4 m/s. We established that the central fuel pin exhibits a slightly higher vibration amplitude than the peripheral fuel pins, which we believe is explained by differences in neighboring environment: the peripheral fuel pins are surrounded by three fuel pins and the immobile test section wall whilst the central fuel pin is surrounded by six mobile fuel pins. We can therefore associate a larger so-called added mass with the peripheral fuel pins [15], resulting in the lower vibration amplitude.

We then approximated the fuel pins as cantilevered beams and discretized classical beam theory to formulate strain-data-dependent displacement equations of the fuel pins according to the study described in [20,21]. These formulations require measurements of bending strain at multiple locations on the fuel pin, which is exactly what we measured with the FBGs. From these equations, we estimated the rms vibration amplitude in terms of displacement taking only bending moments into account. With eight FBGs, the error on the displacement estimation introduced by the model has been determined to be on the order of 10%. The resulting values together with the corresponding standard deviations are shown on the vertical axis on the right-hand side in Figure 6 (left). For sake of clarity, we omitted the additional model error introduced by the beam discretization since these error were significantly smaller that the standard deviation. Note that these values do not describe the complete movement of the fuel pins, as we also identified a rigid body mode corresponding to translation of the fuel (*i.e.*, no bending strain). More details about the nature of this rigid body mode are given in Section 3.2.

Owing to the multiplexing capabilities of the FBGs, we could determine the vibration amplitude with all individual FBGs on a single fuel pin simultaneously. In Figure 6 (right), we show the trend of the vibration amplitude along the central fuel pin. Recall that near the inlet, the fuel pin is supported whilst the other extremity is free to move. Therefore, we observed the highest vibration amplitude (in terms of strain) near the inlet and a decreasing trend in the vibration amplitude towards the outlet. In the figure, we show the rms vibration amplitude determined with the eight FBGs on the central fuel pin. We found that the trend of the vibration amplitude along the fuel pin slightly differs with increasing flow velocity, illustrating the fact that the contributions of the vibration (bending) modes is not constant with flow velocity. The corresponding mode shapes are obtained using a modal analysis which is explained in the subsequent Section 3.2. Note also that although 0.2 μϵ was the smallest detectable strain difference, there were many sources of noise (mainly fluctuating flow conditions) that resulted in a larger spread of derived quantities such as the vibration amplitude. The kurtosis of the resulting vibration amplitude distribution is nearly 3, which illustrates the Gaussian nature of this flow-induced vibration phenomenon.

### 3.2. Modal Analysis Results

Besides the vibration amplitude, the modal characteristics of the fuel pins are also required to assess potential vibration-related safety hazards. To determine the modal characteristics of the fuel pin vibration in the fuel assembly, we used the raw, unfiltered dynamic strain measurements of the FBG sensors as input for the modal analysis. As already discussed in the previous paragraphs, the vibration amplitudes of the pins are very small. Hence, the signal-to-noise ratio is low, which makes it very challenging to obtain reliable from modal analysis results. We therefore applied a state-of-the-art (operational) modal estimation technique, described in [15], which relies on a least-squares estimation in the complex frequency domain [22,23] and which yields a higher accuracy and precision compared to traditional techniques [15]. For sake of completeness, we mention that the estimation procedure assumes a time-invariant, linear system, which is realistic considering that we deal with small vibration amplitudes. We identified modes in the bandwidth from 0.1 Hz up to 200 Hz for flow velocities between approximately 1.3 m/s and 6 m/s.

**Figure 7:**
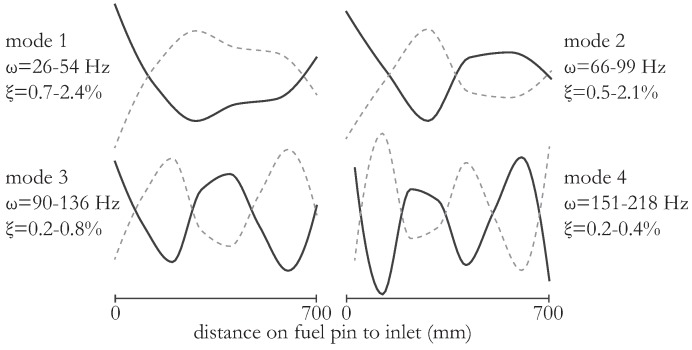
Experimentally estimated mode shapes and approximate eigen-frequency and damping ranges during external excitation of the fuel pins.

The identified eigen-frequencies (ω) and damping ratios (ξ) are listed in Figure 7 for an LBE flow of 2.75 m/s. Note that we identified multiple eigen-modes corresponding to certain flexural modes. This multitude of eigen-modes for each flexural mode is explained by the complex intra fuel pin interaction that is present in an N-pin fuel assembly: for every flexural mode shape of the fuel pins, 2N modes with different cross-sectional patterns exist with slightly different eigen-frequency and damping ratios. We obtained the resulting spread of these modal parameters for the first four flexural modes depicted in Figure 7. As can be seen from the figures, the *strain* mode shapes deviate from classical beam modes for a cantilevered beam, which is due to the tight packing and clustering of the fuel pins. Near the fixation, located leftmost in the figures, the strain amplitude is largest in most cases. Note that, since the excitation forces are unknown, the mode shapes cannot be scaled and have therefore been normalized to unity. However, the combined contribution of all occurring vibration bending modes corresponds to the distribution illustrated in Figure 6 (right). The obtained vibration amplitude combined with these modal parameters are particularly interesting when investigating for example mechanical fatigue.

Since the flow speed can influence the amount of LBE that is dragged along during the fuel pin vibration, the modal parameters are expected to differ with flow speed [15]. However, we observed no effect of flow velocity on the modal parameter, most likely due to the fact that the uncertainty on the estimation (at these low vibration amplitudes) is more significant than the influence. For example, the mode shapes for different flow speeds are very similar and cannot be distinguished with the described set-up since the expected difference in the mode shape (for flow speeds significantly below the critical flow speed) is as small as 1% (as explained in more detail in [15,24]) and is significantly smaller than the uncertainty on the modal parameter estimation. In addition, the complex interaction between the individual fuel pins further complicates the differentiation of the modes.

**Figure 8:**
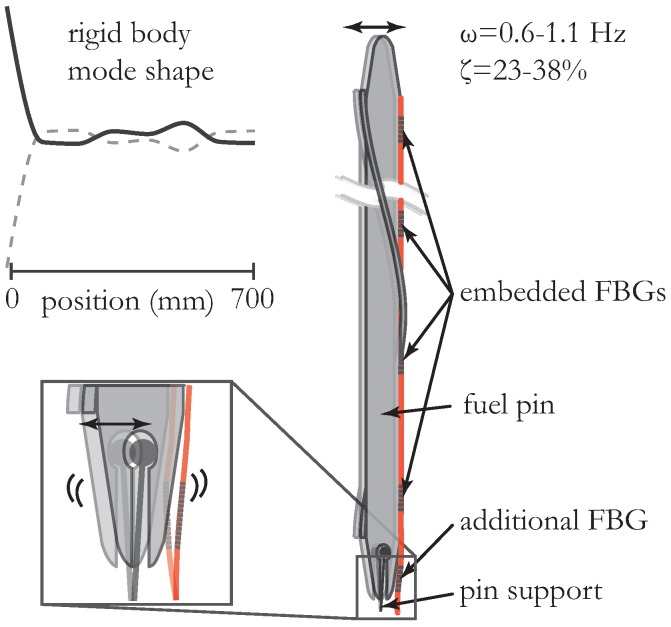
Rigid body “mode shape” occurring as a consequence of the fuel pin fixation or the support structure vibration. The additional FBG located in the capillary measures the relative movement from the fuel pin to its supports. Therefore, only that FBG identifies the rigid body mode.

We also identified a rigid body mode corresponding to the first estimated mode with an eigen-frequency very close to zero. We believe that this frequency corresponds to a rigid-body mode resulting from the allowed movement of the fuel pin supports. In this rigid body mode, the supports or fuel pins oscillate between the limits of their allowed movement at that exact frequency. The rigid body mode is the lateral movement perpendicular to the longest dimension of the fuel pin that stems from the designed fuel pin supports and from the resulting degrees of freedom. This movement along the supports was designed so as to not exceed 1 mm of lateral displacement. The hypothesis of a rigid body mode is confirmed in two ways. First, as can be seen in Figure 8 (left), the damping corresponding to this behavior is large (resulting from the limited allowed movement). Second, the mode shape corresponding to this rigid-body mode agrees with what we expect (also illustrated in Figure 8). Note that a rigid body mode produces no strain in the structure (as a result of bending), but, since we have added a 9th FBG to several fibers (located in the stainless steel tubes close to the fuel pin extremity), we could measure the relative movement between the fuel pin’s extremity and the supports. More precisely, the 9th FBG is strained due to the lateral movement of the fuel pin with respect to its support. Note, however, that the strain measured with this FBG holds information with respect to the rigid body as well as to the bending modes since the bending modes result in a rotation of the fuel pins around their supports. Hence, and because the modes cannot be scaled, the contribution to the measured strain of the rigid body mode is unknown. Nevertheless, the lateral displacement of the rigid body mode is limited by design so as not to exceed 1 mm (see also in the above). With the set-up as described above, we were unable to determine the influence of flow speed on the modal parameters of the rigid body mode, since the rigid body mode is the result of the limited movement of the fuel pins with respect to their supports and no significant effect is expected. The details of this rigid body mode are particularly important in the study of fretting wear [4].

## 4. Conclusions

We have demonstrated, for the first time to the best of our knowledge, the successful use of optical fiber Bragg grating (FBG) based sensors for vibration measurements in a lead-bismuth eutectic (LBE) cooled installation. We first evaluated the vibration amplitude of the fuel assembly during different phases of operation. We then extended our study and used the measured small vibrations to identify modal characteristics of the fuel assembly during operation.

We have shown that the FBGs integrated in the fuel assembly and interrogated as described above allowed measuring local strain levels as small as 0.2 μϵ, which enabled characterizing the vibration of the fuel pins with a sufficiently high precision. We determined the small vibration amplitudes of the individual fuel pins and differentiated based on the fuel pin location in the fuel assembly. In addition, we obtained the distribution of strain along the fuel pins. Furthermore, we applied modal analysis to the high quality vibration data to identify the first five eigen-modes and to estimate the corresponding modal parameters for different cross-sectional patterns of the fuel assembly. To close, owing to the unique properties of FBG based sensors and the application of state-of-the-art modal analysis techniques, we were able to obtain fuel pin vibration characteristics that are important to assess structural degradation resulting from fretting wear or mechanical fatigue and that provide important feedback to the designers of the MYRRHA fuel assembly.
